# Management Guidelines for Diabetic Patients With Hypertension

**DOI:** 10.1111/1753-0407.70093

**Published:** 2025-06-22

**Authors:** Weiqing Wang, Qin Wan, Suijun Wang, Guang Ning, Yufang Bi, Lianyong Liu, Yan Liu, Yu Liu, Xiaoying Li, Tingzhi Li, Xueyi Wu, Shengli Wu, Yubo Sha, Yifei Zhang, Rongyue Chen, Hongting Zheng, Dong Zhao, Ling Hu, Peng Duan, Xinguo Hou, Guoyue Yuan, Yu Xu, Shan Huang, Zhiqiang Kang, Qijuan Dong, Huanfeng Pan

**Affiliations:** ^1^ Department of Endocrine and Metabolic Diseases Ruijin Hospital, Shanghai Jiao Tong University School of Medicine Shanghai China; ^2^ Department of Endocrinology and Metabolism The Affiliated Hospital of Southwest Medical University Luzhou China; ^3^ Department of Endocrinology Shanghai China; ^4^ Department of Endocrinology Punan Hospital of Pudong New District Shanghai China; ^5^ Department of Endocrinology Third People's Hospital of Datong Datong China; ^6^ Department of Endocrinology Sir Run Run Hospital, Nanjing Medical University Nanjing China; ^7^ Department of Endocrinology Zhongshan Hospital, Fudan University Shanghai China; ^8^ Department of Endocrinology Loudi First People's Hospital Loudi China; ^9^ Department of Endocrinology The Second People's Hospital of Guiyang Guiyang China; ^10^ Endocrine and Metabolism Center Karamay Hospital of Traditional Chinese and Western medicine (Karamay People's Hospital) Karamay China; ^11^ Department of Endocrine and Metabolic Diseases The First People's Hospital of Dali Dali China; ^12^ Department of Endocrinology Xuchang Hongyue Diabetes Hospital Xuchang China; ^13^ Department of Endocrinology The Second Affiliated Hospital (Xinqiao Hospital) of Army Medical University Chongqing China; ^14^ Center for Endocrine Metabolism and Immune Diseases Beijing Luhe Hospital, Capital Medical University, Beijing Key Laboratory of Diabetes Research and Care Beijing China; ^15^ The Third Affiliated Hospital of Nanchang University (The First Hospital of Nanchang) Nanchang China; ^16^ Department of Endocrinology and Metabolism The Third Hospital of Nanchang Nanchang China; ^17^ Department of Endocrinology and Metabolism Qilu Hospital of Shandong University Jinan China; ^18^ Department of Endocrinology Affiliated Hospital of Jiangsu University Zhenjiang China; ^19^ Department of Endocrinology Tongren Hospital, Shanghai Jiao Tong University School of Medicine Shanghai China; ^20^ Department of Endocrinology Zhengzhou Central Hospital Zhengzhou China; ^21^ Department of Endocrinology and Metabolism People's Hospital of Zhengzhou Zhengzhou China; ^22^ Department of Endocrinology Jilin People's Hospital Jilin China

**Keywords:** diabetes mellitus, hypertension, medication, risk factor, target

## Abstract

Hypertension is one of the most common comorbidities associated with diabetes. The standardized diagnosis and treatment of hypertension in diabetic patients, along with achieving blood pressure targets, is of great significance for reducing long‐term complications, extending lifespan, and improving quality of life. Recent high‐quality multicenter large‐sample intervention studies have provided important evidence‐based grounds for setting blood pressure targets in diabetic patients. Therefore, based on a comprehensive summary of important domestic and international research literature, and in combination with clinical practice experience, this management guideline is now formulated.

## Graphical abstract

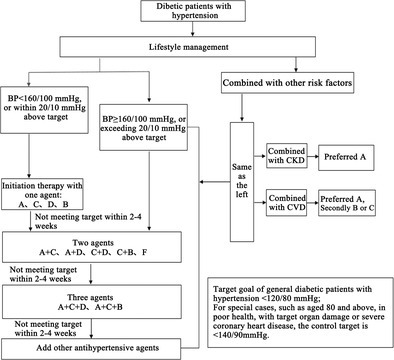

**Summary**

- Hypertension is a prevalent comorbidity in patients with diabetes, necessitating the implementation of standardized diagnostic and therapeutic protocols to mitigate disease‐associated complications and enhance quality of life.
- Recent findings from the Blood Pressure Control Target in Diabetes (BPROAD) study have demonstrated that implementing an intensive systolic blood pressure target of < 120 mmHg resulted in statistically significant reductions in the incidence of major adverse cardiovascular events (MACE) compared to conventional management targeting < 140 mmHg.

## 1 Epidemiology of Diabetes and Hypertension in China

The prevalence of diabetes and prediabetes in China has exhibited a sustained upward trajectory, with the national diabetes prevalence escalating from < 1% in 1980 to 12.4% in 2018. However, among individuals with diabetes, the awareness, treatment, and control rates are notably suboptimal at 36.7%, 32.9%, and 50.1% [1], respectively. Concurrently, hypertension affects approximately 256.7 million adults aged 30–79 years, with an age‐standardized prevalence rate of 27.5% [2]. Data from 2018 reveal critical deficiencies in hypertension management: the “three rates” for hypertension among Chinese adults were recorded at 41.0%, 34.9%, and 11.0%, respectively [3].

Hypertension is a major comorbidity in diabetic patients, with a prevalence of 66.3%, significantly higher than the 21.9% observed in normoglycemic individuals [4]. Alarmingly, blood pressure control rates in diabetic patients remain critically low at 4.7%, significantly lower than the 19.6% observed in normoglycemic controls. Studies indicate that 35% to 75% of cardiovascular risk in diabetic patients is attributable to hypertension, and optimal blood pressure management reduces adverse cardiovascular outcomes in diabetic patients [5, 6]. Therefore, the standardized diagnosis and management of hypertension, along with the attainment of optimal blood pressure targets, are crucial for reducing long‐term complications, extending patient survival, and improving the quality of life in diabetes.

## 2 Risk Factors and Prevention for Diabetes Complicated With Hypertension

Key risk factors include genetic predispositions, aging, obesity, gender, and an unhealthy lifestyle.

### 2.1 High Sodium, Low Potassium, and High‐Fat Diet

Sodium intake is positively correlated with blood pressure, and approximately one‐third of Chinese adults are sodium‐sensitive [7, 8]. Additionally, the International Study of Salt and Blood Pressure (INTERSALT) has proven that potassium intake is also an important factor affecting blood pressure in different populations, with an increased sodium/potassium intake ratio leading to elevated blood pressure [9]. High‐fat diets, particularly those rich in saturated fats, can increase the risk of hypertension [10]. It is recommended to reduce sodium intake and to replace saturated fats (e.g., animal fats, palm oil) with unsaturated fats (e.g., vegetable oils) while avoiding trans fats (e.g., hydrogenated vegetable oils). Research has demonstrated that the Jiangnan diet, similar to the Mediterranean diet, is effective in weight loss, blood glucose control, and blood pressure management [11].

### 2.2 Overweight and Obesity

There is a significant correlation between body weight and the prevalence of hypertension. For every 5 kg/m^2^ increase in body mass index, the risk of hypertension increases by 49%. The prevalence of abdominal obesity in China is as high as 29.1% [12, 13]. Personalized energy‐balance diet plans meeting diverse nutritional needs should be established to achieve or maintain an optimal weight. Exercise also plays a positive role in weight control, improving insulin sensitivity and blood pressure control. Moderate‐intensity aerobic exercise, supplemented by resistance training, balance training, breathing exercises, flexibility, and stretching exercises, are recommended. Intense exercise should be avoided in the elderly [14].

### 2.3 Aging

The prevalence of hypertension increases with age, with over 65 years having a hypertension prevalence exceeding 55%. This is primarily characterized by increased systolic pressure and decreased diastolic pressure, resulting in an increased pulse pressure [3].

### 2.4 Smoking

Smoking is an independent risk factor for cardiovascular disease and is associated with the risk of hypertension and other diseases. Secondhand smoking also increases the risk of cardiovascular disease [15, 16]. Not smoking or having quit smoking for more than 12 months significantly reduces the incidence of cardiovascular complications in individuals with prediabetes and diabetes.

### 2.5 Excessive Alcohol Consumption

Both long‐term excessive alcohol consumption and occasional binge drinking increase the risk of hypertension. The risk of hypertension in male and female drinkers is 1.24 times and 1.41 times that of non‐drinkers, respectively [17]. The risk of hypertension increases with the frequency of alcohol consumption. It is advised that patients limit their alcohol intake and abstain from drinking as much as possible, with a daily intake of alcohol less than 25 g for men and less than 15 g for women, not exceeding 2 times a week [18].

### 2.6 Psychological Stress

Individuals with hypertension are disproportionately susceptible to psychological health issues, as individuals with anxiety disorders exhibit a 1.37‐ to 1.40‐fold higher risk of developing hypertension compared to the general population [19]. A study from China further demonstrated a significant association between psychological stress and hypertension [20]. Compared with the general population, the incidence of depression and anxiety in patients with type 2 diabetes is higher, and such negative emotional states may exacerbate the progression of diabetes mellitus. Additionally, psychological stress and emotional tension in hypertensive patients may exacerbate hypertension‐related symptoms [21]. Mental health is a pivotal component of chronic disease management. Timely recognition and mitigation of depressive and anxious symptoms in patients can significantly enhance quality of life and improve disease management outcomes.

### 2.7 Insufficient Sleep

A U‐shaped relationship exists between sleep duration and hypertension prevalence, with the lowest risk observed at around 6–7 h of sleep per night [22]. Insufficient sleep or poor sleep quality may lead to abnormal sympathetic nerve excitability, causing elevated blood pressure and disruption of the circadian rhythm of blood pressure. Patients are advised to avoid late‐night activities, cultivate regular sleep–wake cycles, and prioritize both the duration and quality of their sleep.

### 2.8 Air Pollution

Both indoor and outdoor air pollution increase the risk of hypertension, with indoor air pollution caused by the use of solid fuels and smoking being related to an increased risk of hypertension [23].

### 2.9 High Altitude

The characteristic climatic features of high‐altitude regions—low atmospheric pressure (hypoxic conditions), cold temperatures, and significant diurnal temperature variations—exert distinct influences on blood pressure. Residents in high‐altitude regions of China exhibit a high prevalence of hypertension, with average blood pressure levels progressively increasing as altitude rises [24].

### 2.10 Other Risk Factors

Other risk factors—including a family history of hypertension, low educational attainment, and lower socioeconomic status—are also associated with an elevated risk of developing hypertension [25, 26].

## 3 Diagnostic Criteria and Standards for Hypertension Measurement

Hypertension is defined as a systolic blood pressure of ≥ 140/90 mmHg measured in clinics on three different days without the use of any antihypertensive medications; or a home‐measured blood pressure of ≥ 135/85 mmHg measured over 5–7 consecutive days; or average blood pressure reading from a 24‐h monitor ≥ 130/80 mmHg, with daytime readings ≥ 135/85 mmHg and nighttime readings ≥ 120/70 mmHg. Patients with a history of hypertension who are currently on antihypertensive medications should still be diagnosed with hypertension even if their blood pressure is below the above diagnostic thresholds [2].

### 3.1 Pre‐Measurement Preparation

Subjects should rest quietly for a minimum of 5 min to relax.

### 3.2 Measurement Posture

The upper arm should be positioned at heart level.

### 3.3 Initial Measurement

On the first visit, blood pressure should be measured in both upper arms, and the reading from the higher side should be recorded.

### 3.4 Repeated Measurement

After an interval of 30 to 60 s, repeat the measurement and take the average of the two readings.

### 3.5 Addressing Reading Discrepancies

If the difference in systolic or diastolic blood pressure between the two readings exceeds 10 mmHg, a third measurement should be conducted, and the average of the three readings should be used.

### 3.6 Measurement in Special Populations

For diabetic patients with hypertension, the elderly, or those with symptoms of orthostatic hypotension, both seated and standing blood pressure measurements should be taken.

### 3.7 Standing Blood Pressure Measurement

Standing blood pressure should be measured at 1 min and 3 min after the subject transitions from a supine or sitting position to standing, and the average of these two readings should be recorded.

## 4 Targets of Blood Pressure Management

When setting blood pressure goals, it is crucial to take into account a person's age, overall health, and any damage to vital organs.

### 4.1 Aiming for a Blood Pressure of Less Than 120/80 mmHg

Current guidelines recommend an antihypertensive target of < 130/80 mmHg for most diabetic patients with hypertension [27]. However, previous studies have demonstrated that when compared to a target of < 140 mmHg, reducing SBP to < 120 mmHg in hypertensive patients significantly lowers the risk of cardiovascular events and all‐cause mortality [28]. The Blood Pressure Control Target in Diabetes (BPROAD) study offers direct evidence for SBP goals in individuals with type 2 diabetes. Findings revealed that in patients with T2DM and hypertension, an intensive antihypertensive strategy targeting SBP < 120 mmHg, compared to a conventional strategy targeting SBP < 140 mmHg, significantly reduces major cardiovascular risks [29]. Given this, under close monitoring for hypotension and electrolyte imbalances during multi‐drug antihypertensive therapy, it is recommended to lower SBP to < 120 mmHg whenever feasible.

During treatment, blood pressure should be rigorously monitored, particularly during initiation and dose adjustment phases. Patients are advised to remain vigilant for symptoms such as dizziness, fatigue, and palpitations, perform regular home‐based blood pressure measurements, and maintain a log to facilitate timely dose optimization by clinicians. In elderly patients, blood pressure monitoring during postural changes is critical, avoiding hypotension from rapid standing. Concurrently, regular electrolyte monitoring is essential, especially when using diuretics, angiotensin‐converting enzyme inhibitors (ACEIs), angiotensin II receptor blockers (ARBs), or angiotensin receptor neprilysin inhibitors (ARNIs). For patients at high risk of hyperkalemia, cautious monitoring and treatment adjustments based on serum potassium levels are warranted.

### 4.2 Targeting a Blood Pressure of Less Than 140/90 mmHg

In certain cases, such as advanced age (specifically ≥ 80 years), poor clinical status, preexisting target organ damage, or severe coronary heart disease, intensive blood pressure lowering may result in serious adverse outcomes due to insufficient perfusion pressure to vital organs. In these cases, it may be prudent to set a less aggressive target of < 140/90 mmHg [30, 31]. This underscores the necessity of tailoring blood pressure goals to the individual, weighing the potential benefits against the risks for each patient.

## 5 Optimal Pharmaceutical Strategies

### 5.1 Antidiabetic Medications

The antihyperglycemic treatment algorithms for diabetes mellitus can be referenced in the guideline for the prevention and treatment of type 2 diabetes mellitus in China (2020 edition) [32] and Metabolic Disease Management Guideline for National Metabolic Management Center (2nd edition) [30]. For diabetic patients with hypertension, it is recommended to prioritize agents with cardiorenal protective benefits.

#### 5.1.1 Agents With Cardiorenal Protective Benefits

Sodium‐glucose cotransporter 2 inhibitors (SGLT2i): SGLT2i reduce blood pressure, cardiovascular events, mortality risk, and heart failure hospitalization in patients with type 2 diabetes [33, 34]. For T2DM patients with atherosclerotic cardiovascular disease (ASCVD) or high‐risk factors, SGLT2i should be prioritized as first‐line therapy unless contraindicated. SGLT2i should also be initiated and maintained throughout treatment in patients with heart failure. Furthermore, SGLT2i significantly reduces the risk of composite renal endpoints [34]. In T2D patients with CKD, SGLT2i with proven renal benefits (e.g., empagliflozin, dapagliflozin) should be prioritized regardless of glycemic status or metformin use, provided the estimated glomerular filtration rate (eGFR) is ≥ 30 mL/min/1.73 m^2^ [27].

Glucagon‐like peptide 1 receptor agonists (GLP‐1RA): GLP‐1RA, including liraglutide, dulaglutide, exenatide, and semaglutide, are cardiovascular and cerebrovascular safe without increasing the risk of hospitalization for heart failure. Notably, liraglutide, dulaglutide, and semaglutide are further associated with reduced risks of cardiovascular mortality [35, 36, 37]. For diabetic patients with ASCVD or high‐risk factors, GLP‐1RA can be employed as first‐line therapeutic agents. Moreover, studies indicate that liraglutide, dulaglutide, and semaglutide significantly reduce renal composite endpoints compared to placebo. These agents represent a viable alternative in patients intolerant to SGLT2i [38, 39, 40].

Metformin: Metformin exhibits cardiovascular‐protective properties. The UK Prospective Diabetes Study (UKPDS) [33] demonstrated its efficacy in reducing the risk of myocardial infarction and all‐cause mortality in obese type 2 diabetes patients [41]. Similarly, the study on the prognosis and effect of antidiabetic drugs on type 2 diabetes mellitus with coronary artery disease (SPREAD) [34] further confirmed that, in Chinese T2D patients with coronary artery disease, metformin significantly lowers cardiovascular event risks compared to glipizide [42].

#### 5.1.2 Other Antidiabetic Drugs

Acarbose was demonstrated to prevent progression from impaired glucose tolerance (IGT) to T2DM [43]. A multicenter prospective intervention study confirmed the efficacy and safety of acarbose as initial monotherapy in Chinese patients with T2DM [44]. Furthermore, a systematic review of clinical studies involving T2DM populations, including Chinese cohorts, demonstrated that α‐glucosidase inhibitors reduce hemoglobin A1C (HbA1C) by 0.50% and promote weight loss [45].

#### 5.1.3 Dipeptidyl Peptidase‐4 Inhibitors (DPP‐4i)

For patients unsuitable for SGLT2i or GLP‐1 RA, DPP‐4i serve as alternative agents with a low risk of hypoglycemia. However, they are not recommended as first‐line therapy due to their relatively limited cardioprotective effects. DPP‐4i are typically combined with metformin or SGLT2i to target multiple pathophysiological pathways of diabetes, offering synergistic glycemic control, potential cardiorenal benefits, and preservation of pancreatic β‐cell function, making them suitable for most T2DM patients.

Other Antihyperglycemic Agents: Additional classes include thiazolidinediones (TZDs), sulfonylureas, and meglitinides, etc. The recently approved glucokinase activators (GKA) have shown promise in improving postprandial glucose levels, though their clinical application prospects require further exploration and validation.

#### 5.1.4 Weight Management in Diabetes and Hypertension

Overweight and obesity are important risk factors for comorbid hypertension in T2DM. Beyond lifestyle interventions, pharmacotherapy should be incorporated with weight‐reducing agents, such as metformin, SGLT2i, and GLP‐1 RAs. These drugs not only optimize glycemic control but also facilitate weight loss, thereby improving blood pressure control.

### 5.2 Antihypertensive Medications

**FIGURE 1:**
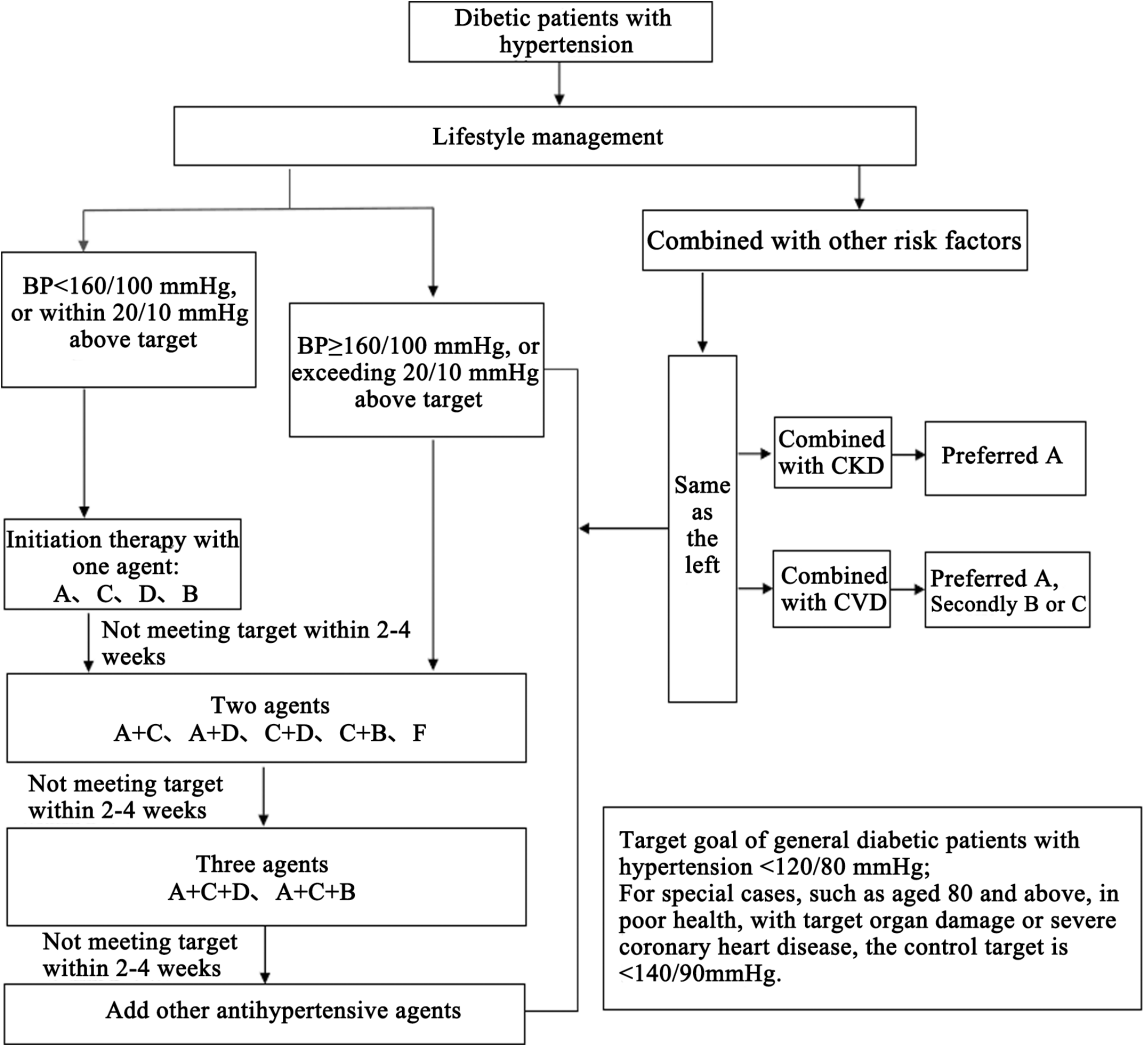
Treatment pathways and drug choices for diabetic patients with hypertension. BP, blood pressure; A, angiotensin‐converting enzyme inhibitors (ACEI) / angiotensin II receptor blockers (ARB) / angiotensin receptor neprilysin inhibitors (ARNI); B, β‐blockers; C, dihydropyridine calcium channel blockers; D, thiazide diuretics; F, fixed‐Dose combination (FDC); CKD, chronic kidney disease; CV, cardiovascular disease.

Prior to initiating antihypertensive therapy, it is crucial to assess target organ damage. If blood pressure remains uncontrolled after lifestyle modifications, pharmacological intervention should be initiated. The selection of antihypertensive drugs should take into account the drugs' efficacy, target organ protection, and safety profile. Refer to Figure 1 for detailed treatment pathways and drug classes.

#### 5.2.1 ACEI, ARB, or ARNI

##### 5.2.1.1 Efficacy and Target Organ Protection

These medications offer cardiorenal protection, reducing the incidence of cardiovascular complications in patients with prior cardiovascular disease and lowering cardiovascular risk in hypertensive patients. They also decrease proteinuria and microalbuminuria in patients with diabetes or chronic kidney disease [46, 47, 48].

##### 5.2.1.2 Preferred Populations for Clinical Benefit

Particularly beneficial for patients with chronic heart failure, post‐myocardial infarction heart dysfunction, atrial fibrillation, diabetic/non‐diabetic nephropathy, metabolic syndrome, or proteinuria/microalbuminuria [21, 49].

##### 5.2.1.3 Adverse Effects and Contraindications

The most common early side effect associated with ACEIs is coughing. Patients with mild symptoms can often continue therapy, but those who are intolerant may switch to ARBs or ARNIs. Prolonged use of those agents may result in hyperkalemia, making regular monitoring of serum potassium and creatinine levels essential. Contraindications include bilateral renal artery stenosis, hyperkalemia, and pregnancy [21].

#### 5.2.2 Calcium Channel Blockers (CCB)

##### 5.2.2.1 Efficacy and Organ Protection

Including both dihydropyridine and non‐dihydropyridine CCB. Dihydropyridine CCB significantly reduces stroke in hypertensive patients [50].

##### 5.2.2.2 Preferred Populations for Clinical Benefit

Particularly suitable for elderly hypertension, isolated systolic hypertension, stable angina, coronary/carotid atherosclerosis, or peripheral vascular disease patients.

##### 5.2.2.3 Adverse Effects and Contraindications

Common adverse effects include sinus tachycardia, facial flushing, ankle edema, and gingival hyperplasia. Patients with tachycardia and heart failure should be cautious when using dihydropyridine CCB.

#### 5.2.3 Diuretics

##### 5.2.3.1 Efficacy and Organ Protection

Thiazide diuretics, such as indapamide, are widely used and have been shown to significantly reduce the risk of recurrent stroke. Potassium‐sparing diuretics, such as amiloride, and aldosterone receptor antagonists, like spironolactone, are effective options for managing resistant hypertension.

##### 5.2.3.2 Preferred Populations for Clinical Benefit

Diuretics are particularly suitable for elderly patients with hypertension, those with isolated systolic hypertension, or individuals with heart failure. They are also a cornerstone therapy for managing resistant hypertension [51].

##### 5.2.3.3 Adverse Effects and Contraindications

Adverse effects are dose‐dependent. Thus, low‐dose therapy is generally recommended. Thiazide diuretics may lead to hypokalemia, necessitating regular monitoring of serum potassium levels and possible supplementation. They are contraindicated in patients with gout.

#### 5.2.4 β‐blockers

##### 5.2.4.1 Efficacy and Organ Protection

β‐blockers are particularly effective in patients with sinus tachycardia and are widely used in those with prior myocardial infarction or chronic heart failure. They also improve prognosis and reduce the risk of cardiovascular events [52].

##### 5.2.4.2 Preferred Populations for Clinical Benefit

Ideal candidates for β‐blocker therapy include patients with tachycardia, coronary artery disease, chronic heart failure, increased sympathetic activity, or hyperdynamic states [53].

##### 5.2.4.3 Adverse Effects and Contraindications

Common adverse effects include fatigue, cold extremities, restlessness, gastrointestinal disturbances, and potential impacts on glucose and lipid metabolism. β‐blockers are contraindicated in patients with second−/third‐degree atrioventricular block and those with asthma. Caution is advised when prescribing to patients with chronic obstructive pulmonary disease (COPD), athletes, peripheral vascular disease, or IGT.

#### 5.2.5 Other Antihypertensive Drugs

Other antihypertensive drugs include α‐blockers, renin inhibitors, etc.

## 6 Key Management Insights

### 6.1 Prospective Multicenter Large‐Sample Evidence‐Based Study—BPROAD Study

The BPROAD study is a randomized, open‐label, outcome‐assessor‐blinded, parallel‐group clinical trial. It enrolled 12,821 patients aged ≥ 50 years with T2D, elevated systolic blood pressure, and increased cardiovascular risk from 145 research centers across 83 cities in 25 provinces/municipalities in mainland China. Participants were randomly assigned to either an intensive‐treatment group (target SBP < 120 mmHg, *n* = 6414) or a standard‐treatment group (target SBP < 140 mmHg, *n* = 6407), with interventions lasting up to 5 years.

The primary outcome was a composite of nonfatal stroke, nonfatal myocardial infarction, treatment or hospitalization for heart failure, or death from cardiovascular causes. At 1 year post‐intervention, the intensive‐treatment group achieved an average SBP of 121.6 mmHg (median: 118.3 mmHg), while the standard‐treatment group reached an average of 133.2 mmHg (median 135.0 mmHg). After a median follow‐up of 4.2 years, the study demonstrated that, under rigorous monitoring for hypotension and electrolyte imbalances, intensive SBP management significantly reduced the incidence of major adverse cardiovascular events (MACE) in patients with T2DM and hypertension compared to conventional SBP management, with a 21% relative risk reduction in the primary composite cardiovascular endpoint.

The BPROAD study is the first to confirm that intensive antihypertensive therapy is superior to conventional antihypertensive therapy in reducing the risk of major cardiovascular events in patients with type 2 diabetes and hypertension. It provides robust evidence supporting intensive systolic blood pressure control in diabetic patients for the prevention of cardiovascular complications. Furthermore, it represents a pivotal contribution to improving cardiovascular health outcomes among individuals with T2DM both in China and globally.

### 6.2 Real‐World Management Experience—MMC Model

Launched in 2016 by Academician Ning Guang and managed by Shanghai National Clinical Research Center for Metabolic Diseases/Shanghai Institute of Endocrine and Metabolic Diseases, the National Metabolic Management Center (MMC) project has been implemented and promoted nationwide. Centered on its core principles of “one center, one stop, one standard model”, MMC integrates standardized diagnostic and treatment protocols with advanced diagnostic and therapeutic technologies, combining the Internet of Things (IoT) and internet‐based platforms to achieve a multi‐beneficial care model that merges online and offline services with hospital‐internal and external connectivity.

Currently led by the Ruijin MMC headquarters, this innovative model has been implemented in over 2000 hospitals across 32 provinces/autonomous regions/municipalities (including Macao, China). To date, the program has managed approximately 2.7 million diabetic patients, with this number continuing to grow. Furthermore, MMC employs a dual‐approach education system for metabolic disease patients, combining in‐ and out‐of‐hospital education programs. It promotes a “Seven Essentials” framework for home management of metabolic disorders, including [30]:

① Regular health education sessions.
② Adoption of balanced nutrition and dietary habits.
③ Engagement in appropriate physical activities.
④ Strict adherence to prescribed medication regimens.
⑤ Home‐based monitoring of blood glucose and blood pressure.
⑥ Maintenance of regular sleep schedules.
⑦ Attention to mental health awareness.

Through the MMC management platform, healthcare providers and patients are effectively supported in achieving optimized disease management. Significant improvements in patients' lifestyle modifications have been documented under this program.

## Disclosure

The authors have nothing to report.

## Conflicts of Interest

The authors declare no conflicts of interest.
